# Treatment of periodontal disease: does drug delivery matter?

**DOI:** 10.3389/fbioe.2024.1427758

**Published:** 2024-07-16

**Authors:** Tarcílio Lima de Sousa, Douglas Dourado, Júlia Soares Rodrigues, Juliana de Souza Rebouças, Marcos Antônio Japiassú Resende Montes, Fabio Rocha Formiga

**Affiliations:** ^1^ Postgraduate Program in Dentistry, School of Dentistry of Pernambuco, University of Pernambuco (UPE), Recife, Brazil; ^2^ Aggeu Magalhães Institute, Oswaldo Cruz Foundation (FIOCRUZ), Recife, Brazil; ^3^ Postgraduate Program in Applied Cellular and Molecular Biology, Institute of Biological Sciences, University of Pernambuco (UPE), Recife, Brazil; ^4^ Faculty of Medical Sciences (FCM), University of Pernambuco (UPE), Recife, Brazil

**Keywords:** periodontal therapy, oral cavity, drug delivery, nanotechnology, biomaterial

## Abstract

**Figure:**
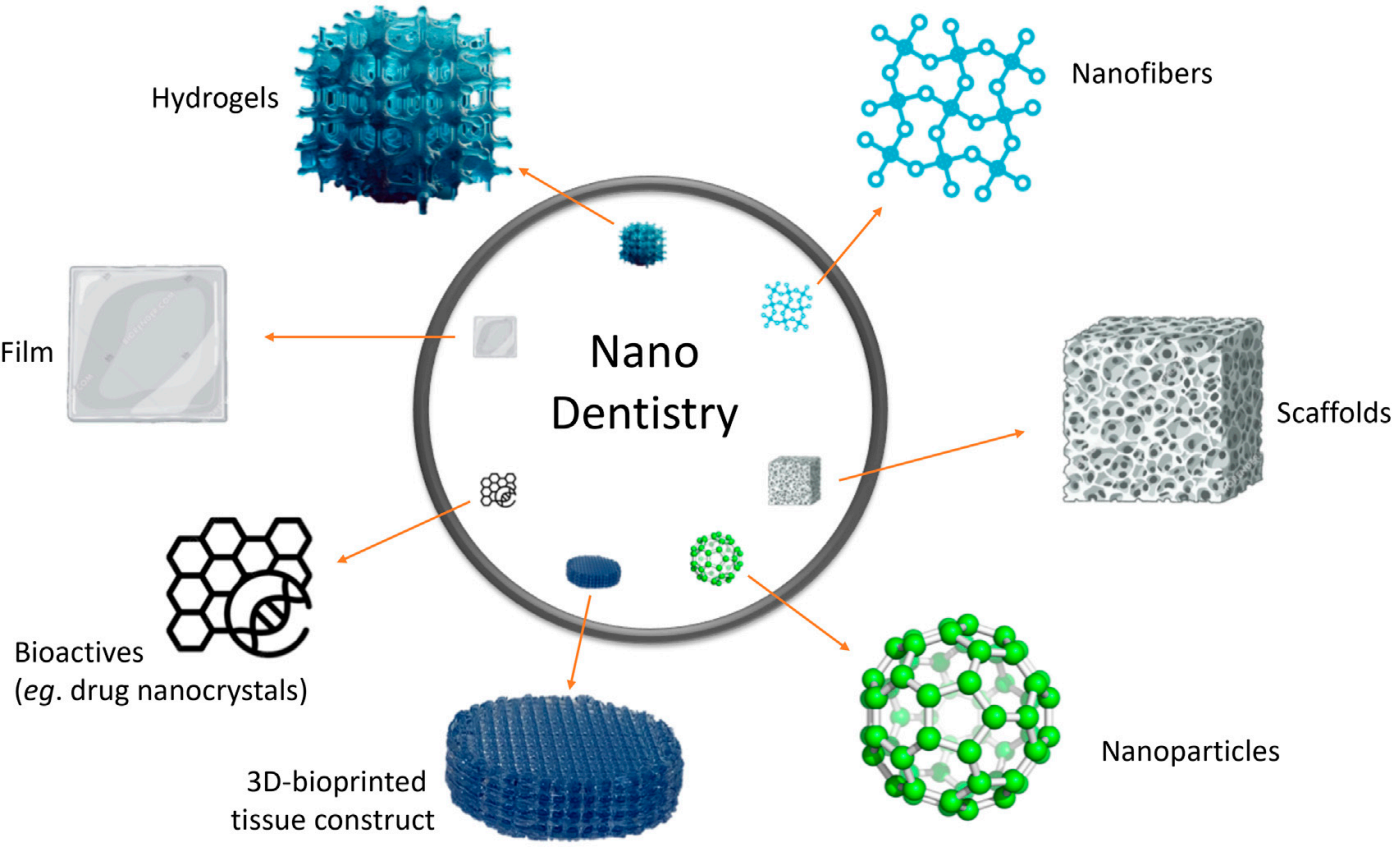

Periodontal diseases (PD) are inflammatory conditions that affect the periodontium, the tooth-supporting apparatus, which includes the gingival tissue, alveolar bone, cementum, and periodontal ligament (Gasner and Schure, 2023). The pathological primary features include clinical attachment loss, radiographically assessed alveolar bone loss, presence of periodontal pockets and bleeding (Papapanou et al., 2018). Gingivitis is a common and mild form of PD; it is a reactive condition that is reversible with appropriate oral hygiene. In turn, periodontitis is an advanced form of PD, when gingivitis has not been adequately treated, leading to a chronic, destructive, and irreversible inflammatory condition. Chronic periodontitis mostly affects adults, but severe periodontitis may occasionally occur in children. Periodontitis is triggered by dysbiosis of the commensal oral microbiota, which interacts with the host’s defense mechanisms, leading to inflammation and disease (Kinane et al., 2017). In fact, periodontitis is initiated by the accumulation of bacterial biofilm, but the underlying mechanisms drive the dysbiosis leading to the dysregulation of the inflammatory response are not completely understood (Bezerra et al., 2022; Abdulkareem et al., 2023).

PD are a major public health problem with high prevalence and morbidity, associated to impaired masticatory function, dental aesthetics and significant dental care costs (Tonetti et al., 2018; Sanz et al., 2020). These conditions affect up to 90% of the global population, making it the most common oral disease. In the United States, nearly 50% of adults aged 30 years and older have some form of PD, and up to 80% of adults have experienced some form of PD in their life. An evidence-based projection in the United Kingdom showed that the adult population with periodontal pocketing is estimated to increase from 25.7 million in 2020 to 27.9 million by 2050. In addition, individuals with tissue attachment loss are projected to increase from 18.6 million in 2020 to 20.9 million by 2050 (Elamin and Ansah, 2023). Overall, PD are more common in men than women, those living in poverty, those with less than a high school education, and heavy smokers (CDC, 2024). As a result, PD are also associated with social inequality, affecting quality of life mainly of vulnerable populations (Velázquez-Cayón et al., 2023).

PD is diagnosed by reviewing the patient’s dental history to identify risk factors, and then comparing these findings with a healthy mouth and normal periodontium. In addition, examining the oral cavity to look for plaque and to check for bleeding can help diagnose PD. Other warning signs include red or swollen gums, painful chewing, sensitive teeth, exudate, gums that have pulled away from the teeth, and loose teeth, among others. A dentist can measure the depth of the pockets between the gums and teeth; in a healthy mouth, it is usually 1–3 mm. Conversely, pockets deeper than 4 mm may indicate periodontitis. Importantly, dental X-rays can be taken to check for bone loss in regions with deeper pockets.

Once PD is diagnosed, it is managed by treating the risk factors. Among them, poor oral hygiene is a key initiator of PD, and preventive practices involve good self-performed oral hygiene. Regular dental checkups are recommended depending on the individual patient’s risk. Tobacco smoking is another important risk factor that is associated with an increased risk of developing PD as well as more severe forms of PD. Moreover, patients who smoke have a significantly lower response to therapeutic interventions against PD (Gasner and Schure, 2023). In addition, diabetes mellitus has a well-documented relationship to PD, and poor glycemic control is linked to increased disease progression (Stoicescu et al., 2021). Other factors that increase the risk of PD include stress, genetics, poor nutrition, obesity, crooked teeth, immunodeficiencies (such as leukemia, human immunodeficiency virus [HIV]/acquired immunodeficiency syndrome [AIDS], and cancer treatment), medications that cause dry mouth, bridges that no longer fit properly, and hormonal changes in women (such as those related to pregnancy, the use of oral contraceptives, or menopause) (Madiba and Bhayat, 2018; Castro et al., 2021).

PD are usually prevented and managed by lifestyle changes and non-pharmacologic therapies, which help to reduce or prevent periodontitis. However, when the disease does not respond to these measures, drug treatment can be administered both locally and systemically, depending on the severity of the illness. Chlorhexidine gluconate is a common antimicrobial compound that disrupts the bacterial cell membrane, increasing the permeability and resulting in cell lysis. Chlorhexidine is generally administered as a mouth rinse, gel, and varnish, and it plays a key role in dentistry to treat or prevent PD (Thangavelu et al., 2020). However, unintentional ingestion of chlorhexidine can lead to adverse effects and systemic toxicity (Kolahi et al., 2006). Another adjunctive antimicrobial treatment is based on local minocycline formulations delivered during periodontal flap surgery and the postoperative maintenance period to treat generalized chronic periodontitis (Abbas et al., 2016). Systemic antibiotic therapy is indicated in patients with persistent deep periodontal pockets and/or with a range of susceptible microorganisms such as *Porphyromonas gingivalis*, *Fusobacterium nucleatum*, *Aggregatibacter actinomycetemcomitans*, *Treponema denticola*, and *Bacteroides* spp. Most of these species exhibit antibacterial resistance. The commonly prescribed antimicrobial drugs include tetracyclines, penicillins, macrolides, quinolones, cephalosporins, and nitroimidazole compounds (Hammami and Nasri, 2021; Haque et al., 2022). Another approach that has been investigated is the use of natural products, which can act as antimicrobials, inhibit osteoclast differentiation, and inhibit the expression of pro-inflammatory cytokines, thus suppressing bone resorption in individuals with periodontitis (Inagaki et al., 2021; Pytko-Polończyk et al., 2021; Song et al., 2021).

Oral antibiotics and anti-inflammatory drugs that are distributed systemically have the potential to induce unwanted side effects, and the development of antimicrobial resistance. Thus, medications administered locally can lead to more positive outcomes in the treatment of PD. Despite the relative success with conservative options, the topical application of drugs to the oral cavity involves some limitations from a drug delivery standpoint. Many anti-biofilm drugs lack efficacy within the oral cavity due to poor solubility, retention, and penetration into biofilms (Sims et al., 2020). There is no ideal approach to deliver drugs into the periodontal pocket and the periapical site. In addition, the oral environment presents chemical and physical conditions that affect the stability, solubility, release, and targeting of compounds used to treat PD (Zhang Y. et al., 2022). In particular, the complexity of bacterial biofilm can modulate the efficacy of drugs in the oral cavity. Biofilms usually reduce the action of drugs on microorganisms, and antibacterial agents are easily metabolized (Rath et al., 2021; Mirghani et al., 2022). Moreover, the salivary flow in the mouth can alter the effect of anti-PD drugs, whereas salivary clearance can dilute these drugs, reducing their local concentrations (Zhang Y. et al., 2022). Therefore, local drug delivery approaches should be considered to optimize the pharmacological intervention used for individuals with PD.

Overall, drug delivery systems can offer diverse benefits, including the ability to increase drug solubility, to control release, to prolong the duration of action, to improve drug targeting, and to reduce cytotoxicity (Ezike et al., 2023). These advantages can overcome the difficulties associated with drug delivery to the oral cavity. Since the late 1990s, several drug-delivery systems have been assessed regarding their functional characteristics, effectiveness, and feasibility for local administration of drugs in the oral cavity (Greenstein and Polson, 1998). In recent years, there has been a growing interest in improving formulations and devices, including tetracycline fibers (Jambhekar et al., 2023), metronidazole and minocycline gels (Ruan et al., 2016; Hasan et al., 2020), minocycline microspheres (Laza et al., 2022), a subgingival chlorhexidine chip (Lecic et al., 2016; Rosa et al., 2021), and doxycycline polymer (Carvalho et al., 2019).

A new generation of drug delivery systems for the treatment of PD includes nanomedicines, which promote local drug delivery to tissues, cells, or subcellular compartments in periodontal pockets. Nanomedicines allow delivery of drugs to biofilm pathogens or host cells, and can control the release of incorporated drugs, usually antibiotics or anti-inflammatory agents. Nanomaterials intended for this application include nanoparticles, nanofibers, nanoemulsions, nanocrystals, liposomes, hydrogels, and films, among others. Studies have shown their potential for the treatment of periodontitis, with enhanced therapeutic outcomes via intra-periodontal pocket delivery (Zupancic et al., 2015; Zhang Y. et al., 2022; Kumaar and Nair, 2023; Zhang et al., 2023). The versatility of these nanomaterials allows drug loading and drug release capabilities to be refined. In addition to their ability to deliver antibiotics, they could be potentially used for regeneration, periodontal repair, and/or immunomodulation (*e.g.,* nanoscale 3D-bioprinted dental tissue constructs and scaffolds). Figure 1 depicts the main types of nanomaterials with potential use as smart drug delivery systems in dentistry, particularly in the treatment of PD.

**FIGURE 1:**
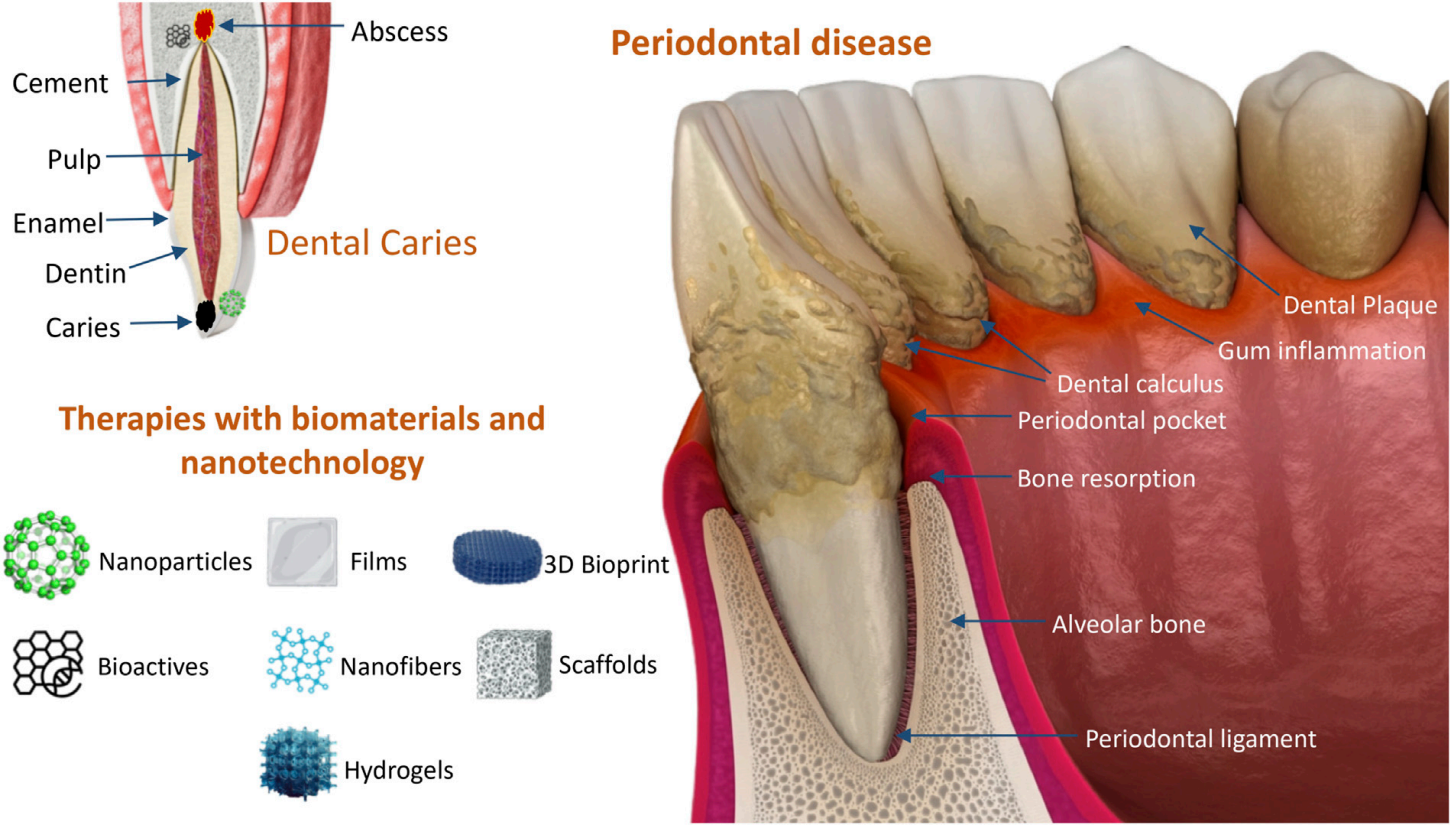
Nanomaterials with potential use as smart drug delivery systems for treatment of periodontal diseases.

In fact, several types of nanoparticles have been investigated as buccal drug-delivery systems for PD, including inorganic and polymer nanoparticles. The shape, size and surface morphology of nanoparticles have been investigated as key parameters related to their favorable outcomes (Bansal et al., 2021). Mesoporous silica nanoparticles (MSNs) have been modified by amination to make them more suitable for use in the oral cavity (Zhang Y. et al., 2022), and to treat white enamel spot lesions (Ren et al., 2023). In another strategy, silver MSNs containing chlorhexidine showed redox/pH-responsive release properties and could inhibit *Streptococcus mutans* biofilm growth (Lu et al., 2018). Metallic nanoparticles containing gold (Ag), copper (Cu), copper oxide (CuO), zinc nanoparticles (ZnONPs), and titanium oxide (TiO_2_) have been investigated as antibacterial agents for many biomedical applications, including potential PD treatments (Nasiri et al., 2023). Ag-TiO_2_ nanocomposites have been synthesized for tailoring the medical grade titanium alloy used for bone/dental implants. This approach resulted in slower silver release, better coverage of the AgNPs on the TiO_2_ nanotubes and strong antibacterial properties (Gunputh et al., 2018). In line with this, Sun and colleagues reported the tetracycline loading on TiO_2_ nanotube surfaces, which showed rapid release of the drug and antibacterial properties (Sun et al., 2018). Overall, despite the potential of inorganic nanoparticles, their biocompatibility and safety require further investigation.

Polymeric nanoparticles have been used in the drug delivery field for a number of diseases, including PD (Basudan, 2022; Bhardwaj and Jangde, 2023). Mahmoud et al., 2019 demonstrated that poly (lactic-co-glycolic acid) (PLGA) nanoparticles modified with BAR peptide inhibited the formation of *P*. *gingivalis* and *Streptococcus gordonii* biofilms in a mouse model of periodontitis. In fact, PLGA nanoparticles have served as efficient carrier systems for several anti-PD drugs, including triclosan (Piñón-Segundo et al., 2005), chlorhexidine (Mosayebzadeh et al., 2023), moxifloxacin (Beg et al., 2020), zinc oxide (Mozaffari et al., 2023), curcumin (Pérez-Pacheco et al., 2021), lovastatin (Lin et al., 2017), minocycline (Zhao et al., 2023), farnesol, and myricetin (Sims et al., 2020), among others. In addition, studies have demonstrated the potential of PLGA nanoparticles to improve the delivery of metformin in an experimental periodontal disease model using diabetic rats (Pereira et al., 2018; Pereira et al., 2021). Beyond PLGA, other polymers have been used to develop drug nanocarriers for PD. For example, chitosan nanoparticles loaded with curcumin exhibited sustained release and anti-inflammatory properties, and showed efficacy in a mouse model of periodontitis complicated with hypertension (Xu et al., 2023). PLGA/chitosan nanoparticles encapsulating metronidazole and *N*-phenacylthiazolium bromide have been assessed as an inflammation-responsive approach to modulate the progression of periodontitis (Lin et al., 2018). In a similar strategy, Chang et al., 2020 demonstrated that core-shell PLGA/chitosan nanospheres containing simvastatin and doxycycline significantly inhibited *P. gingivalis* and *Streptococcus sanguinis*, and promoted the repair of infected periodontal sites in rats.

Other drug carriers such as lipid-based systems have also been investigated for the treatment of oral mucosal infection. Solid lipid nanoparticles (SLN) containing curcumin promoted drug retention in mucosal tissue, indicating preferential mucosal uptake. Moreover, this nanoformulation showed higher antimicrobial activity compared with free drug against *Staphylococcus aureus*, *S*. *mutans*, *Escherichia coli*, *Lactobacillus acidophilus*, and *Candida albicans* (Hazzah et al., 2015). In another study, metronidazole-loaded SLN were incorporated in hydroxyethyl cellulose gel, which exhibited a sustained *in vitro* drug-release pattern, optimal *ex vivo* permeability, and enhanced *in vitro* antimicrobial activity (Ho et al., 2022). In a different strategy, *in situ* nanoemulsion gel was developed as a lipid carrier for intra-pocket drug delivery to treat periodontitis. This approach offered sustained release of azithromycin and showed no toxicity in the hen’s egg test on chorioallantoic membrane (HET-CAM) (Monika et al., 2022).

Liposomes are spherical lipid vesicles composed of one or more lipid bilayers, as a result of emulsifying natural or synthetic lipids in an aqueous medium. Many liposome-based drug-delivery systems have been clinically approved to treat diseases such as fungal and parasitic infections (Petersen et al., 2018). Some researchers have reported on the use of liposomes as drug-delivery systems to treat periodontitis. For example, liposomes stabilized with polyethylene glycol (PEG) and containing magnetite nanoparticles were able to penetrate into dentinal tubules in an *ex vivo* validation experiment performed on extracted human teeth (Di Turi et al., 2012). In addition, liposomes promoted superior delivery of the bactericidal agents triclosan and chlorhexidine, efficiently targeting *Staphylococcus epidermidis* and *S. sanguinis* biofilms (Jones et al., 1997). The major limitation of liposomes as buccal drug-delivery systems is their aggregation with salivary compounds such proline-rich proteins and divalent cations (Ca^2+^ and Mg^2+^). This can be potentially overcome by coating the liposomes with pectin, which has a net negative charge (Nguyen et al., 2013).

In addition to antimicrobial agents, drug delivery strategies for PD can involve the use of growth factors, devices, biomaterials, and tissue-engineering approaches to promote synergistic mechanisms, such as an antibiotic effect, immunomodulation, and tissue repair and regeneration (Kinane et al., 2017). In this sense, mesoporous calcium silicate nanoparticles loaded with gentamicin and fibroblast growth factor 2 (FGF-2) inhibited bacterial viability and showed bone/cementum tissue regeneration, suggesting their use as biocompatible dental pulp tissue regenerative biomaterial (Huang et al., 2017). Chen et al., 2019 developed a thermosensitive hydrogel for sustained co-delivery of ibuprofen and FGF-2. It promoted the proliferation and adhesion of human gingival fibroblasts *in vitro*, thus representing a promising drug-delivery system with the potential to provide early local treatment for peri-implantitis. Other approaches involving growth factor delivery, stem cells, and biomaterial scaffolds have been also reported (Yadlapati et al., 2017; Pilipchuk et al., 2018; Pan et al., 2019; Chen et al., 2022; Mozaffari et al., 2023). In a strategy combining antibacterial properties and dental tissue regeneration, Xu and collaborators added silver and strontium to porous structures made of hydroxyapatite and chitosan, resulting in a biomaterial able to inhibit the growth *S. aureus* (−98%), and promoting osteoconductivity and mineralization (Xu et al., 2016).

Recently, novel alternative devices for delivering antibiotics, cytokines, and other agents to the oral cavity have been investigated. Among them, the use microneedles represents a microscale physical enhancement method that has greatly expanded the spectrum of drugs that can be delivered via different routes, including buccal delivery (Li et al., 2023). A modular microneedle patch containing tetracycline-loaded PLGA nanoparticles demonstrated therapeutic potential for local immunomodulation and periodontal tissue regeneration (Zhang X. et al., 2022). In addition, microneedles carrying metronidazole for controlled periodontal drug delivery showed positive results in a rat model of periodontitis (Song et al., 2023).

In recent years, three-dimensional (3D) bioprinting technology has emerged as a new approach for periodontal regeneration. It allows one to print biocompatible membranes and scaffolds as well as living cells and matrix components in complex 3D functional tissues (Ma et al., 2019). Theodoridis et al. (Theodoridis et al., 2023) developed a scaffold based on poly (ε-caprolactone) containing tetracycline hydrochloride; it promoted antimicrobial activity, an organic collagen matrix, and new mineralized bone tissue.

Despite the research efforts being made on development of nano-based drug delivery systems and biomaterials for PD treatment, not many technologies have advanced from basic to clinical assessment. For example, a mucoadhesive gel containing polymeric ganglioside-coated nanoparticles loaded with satranidazole was evaluated in a 21-days single blind clinical trial. As a result, a greater antibacterial activity was determined, and a decrease in clinical markers of periodontitis, such as gingival index and pocket depth (Singh et al., 2015). Another disease marker, alanine transaminase (ALT), has clinical significance in PD since it might represent the degeneration and inflammation of periodontal tissue. In this context, the impact of ZnONPs on ALT activity was examined in the saliva of 20 patients with chronic PD and 15 healthy volunteers. The results revealed a significant increase of salivary ALT activity in PD patients compared to healthy subjects. Interestingly, there was a significant elevation in the activity of ALT by the effect of ZnONPs in PD patients when compared with PD patients who did not receive ZnONPs, that is mean ZnONPs caused activation on ALT enzyme activity (Talalabd et al., 2017). Moving to biomaterial clinical investigation, in a 4-year clinical study with 20 patients suffering from moderate peri-implantitis, Schwarz et al., 2009 showed that the application of natural bone mineral in combination with a collagen membrane resulted in clinical improvements as compared to nanocrystalline hydroxyapatite.

Besides the lack of consistent clinical studies, additional challenges are related to difficulty in technology transfer to the pharmaceutical market towards PD. This includes a safe translation of laboratory innovations (e.g., cutting-edge nanomedicines and/or smart biomaterials) into commercially viable products for PD therapy. Several concerns such as toxicity of nanomaterials need to be better understood to fully exploit their potential in dentistry (Kunrath et al., 2023). In a certain manner, the interactions of nanomaterials with alveolar bone and gingival mucosa are still little known, and the main mechanism of toxicity seems to be the induction of cellular oxidation, leading to greater production of reactive oxygen species (ROS). Consequently, this could lead to changes in cellular signaling cascades, generating protein radicals and lipid peroxidation, DNA damage, initiating of inflammatory responses and, eventually, cell death (Pokrowiecki et al., 2018). Additionally, a deeper knowledge of these interactions, nanotoxicity assessment, and *in vitro-in vivo* modeling may be evaluated. For that, it is important to consider the raw (nano)material selection, composition, particle size, surface charge and interactions with the host tissue (Sreenivasalu et al., 2022). Therefore, the resolution of these challenges should guide the rational development of nanomaterials and their clinical translation towards PD treatment.

Given all the above, the modern treatment of PD includes the control of the modifiable risk factors, but complete regeneration of the periodontium can be potentially achieved by using drug nanocarriers and/or biomaterials. While there have been substantial advances in this field, researchers continue to develop novel strategies such as those mentioned in this article. The focus should be on exploring ways to improve conventional therapies, such as antibiotics, while also pursuing innovative tools to treat patients with PD and to improve their of quality of life. In this scenario, efforts from multidisciplinary investigations involving dentistry, material science, microbiology, biotechnology, and pharmaceutics have resulted in the development of local drug-delivery systems that are more efficacious and less toxic than previous treatments. Taken together, these investigations have provided a solid scientific basis for drug-delivery systems in PD treatment, from basic research to proof of concept in animal models of periodontitis, showing the potential of nanocarriers for better medication delivery. The major challenge is now to translate the *in vitro* and *in vivo* findings to humans, by resolving concerns such as toxicity profile, scale up manufacturing, and addressing regulatory issues. Moreover, well-conducted clinical trials involving patients with PD are required. Hopefully, the intensive research together with the unmet patient care needs will lead to a promising future with better treatments for PD.
